# Analysis of Public Policies on Food Security for Older Mapuche Adults in Rural Areas

**DOI:** 10.3390/foods14061055

**Published:** 2025-03-19

**Authors:** Angélica Hernández-Moreno, Fernanda Gutiérrez-Gutiérrez, Natalia Celedón-Celis, María Girona-Gamarra, Jorge Hochstetter-Diez

**Affiliations:** 1 Departamento de Salud Pública, Facultad de Medicina, Universidad de La Frontera, Temuco 4780000, Chile; angelica.hernandez@ufrontera.cl (A.H.-M.); natalia.celedon@ufrontera.cl (N.C.-C.); 2 Departamento de Ciencias de la Computación e Informática, Universidad de La Frontera, Temuco 4780000, Chile; fernanda.gutierrez@ufrontera.cl; 3 Departamento de Nutrición Básica, Escuela de Nutrición, Universidad de la República, Montevideo 11600, Uruguay; mgirona@nutricion.edu.uy

**Keywords:** food security, public policies, indigenous communities, older adults, Mapuche, ethnographic analysis

## Abstract

Food security remains a critical challenge for older adults in rural Indigenous communities, particularly among the Mapuche people. This study presents an analysis of public policies that address the food security of Mapuche older adults in rural Chile. Using an interpretative qualitative approach, we explore the alignment between government programs and the lived experiences of this population. Findings indicate that existing policies lack cultural pertinence, focusing primarily on market-driven agricultural production rather than self-sufficiency and traditional food systems. Participants highlight the loss of community farming practices, environmental degradation, and the imposition of external production models as key factors exacerbating food insecurity. In addition, health and education policies do not integrate Indigenous knowledge and food habits, which further limits their effectiveness. This study highlights the need for culturally inclusive public policies that support local food sovereignty, sustainable agricultural practices, and the empowerment of Indigenous communities.

## 1. Introduction

We find ourselves in a complex historical moment at a global level, in which great crises converge and interact: the financial, environmental, and socio-political crises [1,2]. According to several authors, the origin of these crises lies in the capitalist expansion promulgated in 1949 in the United States under the concept of underdevelopment [3,4]. Although this concept integrated the notion of social welfare in its origin, its implementation was related to the emphasis on economic growth [3]. The impact of such policies was clearly expressed in those nations considered to be underdeveloped, in greater poverty and inequality [3]. Subsequently, with the contribution of scientists and academics, notions of development that gradually incorporated other approaches, such as social change, environmental, cultural, human, and human rights, were raised [5,6,7]. However, these notions, in the planning and practice of plans and policies, were formulated from a global perspective, without citizen participation, and with a profound colonialist and capitalist approach [8,9,10,11]. Imposed under an approach of domination and dispossession, and leaving aside the needs of the population, these notions, still in force today, have resulted in the deterioration of these societies and are manifested in the crises we are currently experiencing [3,9,11,12].

Thus, current policies and the institutions that develop them maintain these approaches. Although they have connotations linked to the sociocultural, their reductionist economic approach takes precedence, affecting welfare and localized development in territories [13,14,15]. The agrifood sector does not escape this vision under the bastion of the green revolution [16,17].

The green revolution, amplified since 1939 in most parts of the world, emerged as a solution to the perceived risk of insufficient food production for the sustained population increase [17]. It consisted of the transformation of local food production systems to a package of synthetic chemical inputs, the use of machinery and hybrid, and then genetically modified seeds for intensive monoculture production [17,18]. This system was used homogeneously, without considering local geophysical and capital conditions, reporting good results in the yield of some cereals and generating great benefits for big capital and industrialization but not for small producers. After years of use, rural poverty increased, with loss of biodiversity, soil infertility, and loss of cultural practices for food production [18,19,20] de reproducción social y de consumo de alimentos [21].

This is because agribusiness, due to its high levels of entropy, exerts considerable pressure on the environmental limits of the biosphere [22,23,24]. Its operation depends on extensive areas of land, which favors deforestation and desertification [25,26,27]. It also requires large quantities of water, which are often obtained through misappropriation practices [28,29]. Likewise, the intensive use of agricultural inputs contributes to rapid soil degradation, increasing the need to resort to costly technological investments to maintain its fertility and productivity [24,27,30]. Therefore, from farmers and Indigenous organizations around the world, the inequalities present in production, the food market, and the dynamics of international trade have led to the need to move towards another production system linked to food sovereignty and sustainable production, considering traditional knowledge [31].

The agricultural sector plays a crucial role in food security, as it is often the basis of economic activities in many countries [32,33,34]. Specifically, family farming plays this role, especially in rural areas, where these farms are often the backbone of local economies because of their ability to provide employment, reduce poverty, and promote sustainable agricultural practices, increasing the resilience of communities in the face of food insecurity [35,36,37].

Food insecurity is defined as the lack of access, availability, bioavailability, and stability of food, which are essential dimensions of food security to ensure adequate nutrition [38]. Although the most recent data show that between 2021 and 2022, food insecurity was reduced, progress is insufficient to achieve the SDG 2 (Zero Hunger) targets [39,40]. The world is still dealing with the consequences of the COVID-19 pandemic, climate crisis, and active conflicts, significantly impacting food security figures in Latin America and the Caribbean (LAC) [39,41]. This is further aggravated by market concentration practices, exacerbating prices by restricting competition and influencing costs, affecting consumers and especially regions that are more dependent on food imports [42,43].

By 2023, food insecurity affected 40% of the population in LAC, mainly affecting women, children, and older people, the latter being a severely disadvantaged group [44,45].

The world’s population is estimated to be aging. By 2050, the population of older people is expected to increase to 1.5 billion people, representing 16% of the global population, up from an estimated 9.3% in 2020 [46]. Limited access to resources exacerbates the deprivation of vulnerable groups, factors that are directly related to poverty, lack of health coverage and food security, all risks to human rights [41,47,48].

Ageing-related constraints are key elements in the nutrition and food security of older people [49]. The physiological changes associated with aging, such as sensory, metabolic, gastrointestinal, musculoskeletal, and others, need to be addressed with a preventive approach to maintaining the health and quality of life of older people [50]. Neurodegenerative pathologies can also be addressed preventively. This is where availability and access to healthy nutrition for this age group is essential [51].

The quality of the diet protects this age range from malnutrition in all its forms, as well as from chronic non-communicable diseases [52]. In addition, the condition of aging, especially when combined with an Indigenous and rural context, has been identified as intersectionality that significantly aggravates food insecurity [53,54,55].

Chile has one of the highest levels of inequality and a high cost of food, especially healthy food [56,57]. Rising international food prices and the consumer price index (CPI) for food have only worsened the situation, and it is becoming increasingly difficult for older people to meet the recommendations of the Chilean population’s Food-Based Dietary Guidelines (FBDG). Disproportionately affecting those living in vulnerable conditions (i.e., women, children, Indigenous peoples, older people, and people of African descent) [56,58].

Public policies are key to addressing food security challenges and require a multidisciplinary and holistic approach based on social determinants to address health inequalities at the global level, prioritizing the most disadvantaged socio-economic groups [59,60]. In particular, Chile still has a great deficit in terms of state initiatives, which lack a fully integrated vision [61,62]. One example is the case of Mapuche communities in rural areas, which lack actions aimed at improving food security [63]. It is crucial to adopt a holistic approach in the design of food security-oriented public policies, integrating not only the mitigation of climate change risks but also the improvement of food education, the strengthening of social support networks and sustainable production [64,65].

This study aims to analyze the relevance of public policies related to food security in rural Mapuche older adults from the perspective of the subjects themselves in the year 2023. This approach allows an understanding of the cultural and contextual adequacy of these policies in historically invisible communities. By incorporating the voices of those who experience these realities, the analysis seeks to identify gaps and opportunities for a more inclusive and respectful implementation of their sociocultural particularities. In this way, the study not only broadens the discussion on food security but also proposes strategies adapted to the real needs of vulnerable groups. Likewise, this research is oriented both to the generation of knowledge, contributing to the reduction of information gaps on local contexts and specific groups, and to the promotion of social justice in the face of structural transformations and ethnic prejudices that persist.

## 2. Background

### 2.1. Food Security, Climate Crisis, and Social Inequality

Food security refers to continued access to sufficient, safe, and nutritious food for a healthy life, taking into account dietary needs and cultural preferences [66]. Its measurement, through the Food Insecurity Experience Scale (FIES), indicates the proportion of people experiencing difficulties in obtaining food. Moderate food insecurity involves uncertainty and occasional reductions in food quality or quantity, while severe food insecurity involves episodes of hunger and, in extreme cases, days without food, putting health at risk [39].

Particularly for LAC, food insecurity remains a critical problem [67]. In 2022, it affected 37.5% of the population (247.8 million people), which, although down 2.8 points from the previous year, is high and exceeds the global average (29.6%) [39]. In addition, the prevalence of food insecurity has increased from pre-pandemic levels (27.3% in 2015 and 31.5% in 2019) [39]. In Chile, between 2020 and 2022, moderate or severe food insecurity reached 18.1% of the population (3.5 million people), a significant increase from 10.8% between 2014 and 2016 [39]. Although this percentage is below the regional average (37.5%), it should be taken into account that it disproportionately affects rural and Indigenous groups [68].

The global food market faces problems due to the concentration of large companies that dominate the trade, limiting competition and influencing prices [69,70]. This phenomenon exacerbates prices by influencing costs, affecting consumers and especially regions that are more dependent on food imports [71].

The COVID-19 pandemic, the climate crisis, the conflict in Ukraine, the economic slowdown, and food inflation have severely affected food security in LAC [72,73,74,75]. The impacts of climate change and persistent social inequalities in the region vary significantly among different social groups, influenced by factors such as gender, age, ethnicity, wealth, and social class [76,77]. Women, children, and the elderly face greater challenges due to poverty, socio-economic and political exclusion, their traditional roles in the family and community, and their dependence on activities such as subsistence agriculture, which is highly vulnerable to climate change [39,78,79].

Inequality in food security and nutrition is even more evident between rural, peri-urban, and urban areas due to unequal access to resources such as wealth, education, health, and infrastructure [80,81,82]. Globally, moderate or severe food insecurity is more prevalent in less urbanized areas [39,83]. Particularly in LAC, the gap between rural and urban areas was 8.3% in 2022, exceeding the global average of 7.3% [39].

Through food, individuals not only obtain nutrients that enable them to sustain and reproduce life [84,85]; social groups construct a set of social representations about food that trigger their daily food practices. In particular, Väätämöinen and Merino [86] highlight the role of food as an essential tool for Mapuche immigrant families to reinforce their ethnic identity and transmit it to younger generations in Santiago, Chile. These authors affirm that for this social group, food allows them to stay connected, strengthening feelings of belonging in a physical context that is alien to them. Thus, it is relevant to investigate the interaction between meanings, beliefs, and food culture, focusing on the constructions of collective food meanings and consumer behaviors in a context of food (in) security linked to a damaged food system that causes unequal physical and economic access to food [87,88]. Food security (FS) is an effective social determinant of health (SDH) and is crucial for the health of people and the planet. Being a multidimensional construct that must take into account calories consumed and diet quality, food safety, social, cultural, and health aspects in changing social and political frameworks [89,90].

The current food system has led to a loss of food diversity, environmental degradation, and degradation of people’s diets with serious consequences on health and economic growth, and is affected by multiple sociocultural factors [91,92]. Specifically in the Chilean context, and taking into account some aspects related to FS, Barreau et al. [93] assert that communities that use local foods in Chile are more likely to be food secure over time. In exploring the current and past food systems of families living in Mapuche territory, they found that older participants agreed that food systems have changed a lot throughout their history, as well as the way food is sourced and prepared.

### 2.2. Ageing and Nutrition

The world’s population is aging rapidly, projecting rapid growth in the proportion of older people in the coming decades [46]. Although this phenomenon is more evident in high-income countries, it also affects lower-income nations, where lack of resources exacerbates the deprivation of older adults [94]. In many cases, old age is associated with poverty and lack of access to health services and food, which puts fundamental rights at risk [45,95,96,97].

On the other hand, the public health system, under its biomedical approach, does not develop the protective capacities of the communities’ health. Practices that affect the preservation, care, and recovery of health and that include diverse strategies and knowledge transmitted orally or traditionally within the socio-territorial context of individuals or groups, which respond to the worldview of each community, from which the health-disease process is interpreted, and measures are adopted for its management [98].

Rising food costs, declining physical and mental capacities, and socio-economic factors increase the nutritional vulnerability of older people, affecting their quality of life and aggravating pre-existing health conditions such as chronic non-communicable diseases [99,100,101]. These difficulties are even greater in rural or Indigenous contexts, where aging is closely linked to greater food insecurity [63,102].

### 2.3. Family Farming

Family farming plays a key role in rural areas in strengthening community resilience to food insecurity [36,37,103]. Techniques such as crop diversification and home gardening improve food access and nutrition, reducing vulnerability to climate change and other challenges [37]. However, family farming faces obstacles such as land fragmentation and market failures, which require specific solutions to maximize its impact [104,105].

### 2.4. Gaps in Public Policies

Public policies are essential to address food security challenges, integrating technological, institutional, and governance solutions to achieve the Sustainable Development Goals (SDGs) and reduce hunger and malnutrition [106,107]. Public policies also play a key role in promoting family farming, as demonstrated by the National School Feeding Program (PNAE) in Brazil, which improves the food security and income of small farmers by prioritizing the purchase of local products [108]. In Cameroon, initiatives focusing on access to markets and agricultural inputs strengthen family farming as a pillar of food security [61].

Although Chile has made progress in formulating food security policies, current challenges highlight the need for a more comprehensive strategy that addresses both the reduction of vulnerability of certain groups and the implementation of long-term sustainable practices. While the Ministries of Health, Social Development, Agriculture, and Economy have implemented various policies and programs aimed at improving access to food and supporting local production, such policies often lack an integrated approach for rural areas, as is the case in many Mapuche communities, where food insecurity remains a concern due to structural factors such as limited access to agricultural inputs and markets, and lack of specific adaptation to local realities [63,109].

Although there are initiatives that may indirectly benefit older adults in rural communities, the Programa de Alimentación Complementaria del Adulto Mayor (PACAM) remains the only public policy explicitly focused on the food security of this age group. Implemented by the Chilean Ministry of Health, this program distributes fortified foods to people over 65 years of age as preventive nutritional support and for the recovery of nutritional deficiencies [110,111]. Through primary health care (PHC) establishments, a food supplement enriched with micronutrients, specially designed for their needs, is provided to maintain or improve their physical and mental functionality [110,111]. However, evaluations indicate low adherence to the program, with only 42.2% of beneficiaries correctly consuming the Golden Years Milk Drink, while in the case of Golden Years Cream, the figure drops to 6% [112]. Furthermore, only 4.4% of people consume both products according to the recommended indication [112]. This is why this program was reformulated in 2021 to improve its nutritional quality and acceptability [113].

Other programs, such as the Family Self-Support Program (FOSIS) or the Indigenous Territorial Development Program (PDTI), do not have an exclusive focus on older people [114,115]. Even the Special Program on Health and Indigenous Peoples (PESPI), while incorporating an intercultural approach, does not specifically address food security [116].

## 3. Materials and Methods

Following the methodological guidelines of Denzin and Lincoln [117], this research is part of an interpretative qualitative methodology, which allows us to understand the social reality from the perspective of the participants, exploring their meanings and experiences. Qualitative research is not limited to a single methodological approach but is characterized by its plurality of inquiry strategies and its emphasis on the contextualized interpretation of social phenomena. In this sense, we have opted for a design that integrates methodological tools typical of ethnography, such as the ethnographic interview and participant observation, allowing an in-depth approach to the perceptions and practices of Mapuche elders and leaders of the territory in relation to food security. However, the study does not constitute an ethnography in the strict sense of the term since neither long-term sustained fieldwork nor a prolonged reflective engagement with the community has been developed, aspects that characterize traditional ethnographic research [118]. Instead, an interpretive approach is used that looks at the process from the symbolic interpretive paradigm [119] and that favors interaction with participants and the reconstruction of meanings based on their discourse and daily practices and the complexity approach [120]. Both theoretical positions allow describing and analyzing the subjectivity expressed by people to answer the research question: What is the relevance of public policies related to food security according to the perception of Mapuche people over 60 years old and leaders, inhabitants of the rural area of Temuco in 2023?

**Figure 1:**
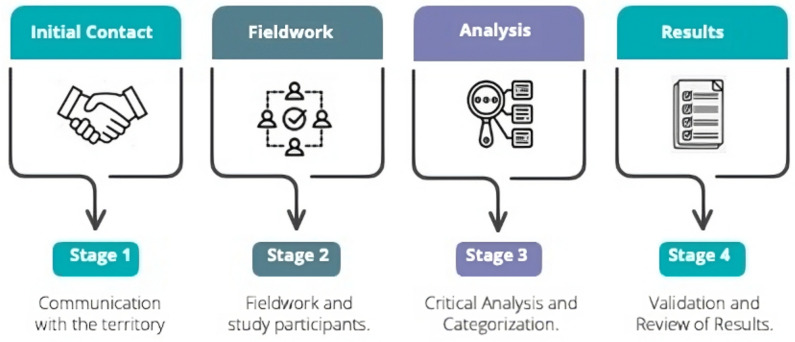
Work methodology.

The research was approved by the Scientific Ethics Committee of the Universidad de La Frontera in Evaluation Act N°075/22, dated 3 August 2022. The fieldwork was carried out using qualitative methodology, integrating ethnographic methodological tools to delve into the practices and perceptions of the participants in relation to food security. Convenience sampling was carried out according to the objectives, which allowed the selection and participation of the inhabitants of the territory. The methodology is divided into four stages (see Figure 1).

(i) Stage 1: Communication with the territory. The channels of trust already established with the Boyeco Territorial Bureau (space for dialogue and coordination between the community and local authorities) were used, who were informed of the project, and the communities to be included in the study were defined. The communities were selected with the support of leaders of the Boyeco Territorial Bureau, with two criteria: geographic spatiality and greater presence of elderly people. For geographic spatiality, the map of the territory was observed and divided into four segments, and with the support of the leaders of the Territorial Bureau, the communities to be intervened in each segment were identified, as well as the contact with their leaders. Once contact was established with each community, the elderly were recruited in two ways: by participating in community meetings, explaining the project, and inviting them to participate, and by direct contact between community leaders and older adults.
(ii) Stage 2: Fieldwork and study participants. This stage took place in nine Mapuche communities in Boyeco, Temuco, in the Araucanía Region of Chile. The interviewees included both men and women over 60 years of age, as well as leaders of the aforementioned territory. An ethnographic interview was used as an instrument, adapted to each subject and their context, and guided by the research objectives. The strength of this type of interview lies in its flexibility and the possibility it offers participants to express their ideas clearly and in-depth. For this reason, the questions are based on conceptual themes suggested by the objectives, without asking closed or repetitive questions for each interviewee. Eleven open-ended interviews were conducted, with an average duration of one and a half hours each. As a triangulation technique, two focus groups were organized, each with ten participants—men and women—all members of community organizations. The sessions lasted approximately two hours each. Both instruments were applied in different communities in the same territory, and were complemented with field notes recorded in the process of participant observation, both during the application of techniques in homes and community centers, as well as in assemblies and community meetings in which we participated.
(iii) Stage 3: Analysis of results. To obtain the results, an intersectional analysis of the data was carried out, based on grounded theory [28], supported by the Atlas.ti 22 program. This process relates the research objectives with the discourse obtained through the techniques used, selecting key quotes. From the interviewees’ responses, emerging conceptual categories were identified that reflect their particular meanings and worldviews, which provide answers to the research objectives. Subsequently, more abstract ideas associated with existing theory were developed, building conceptual networks in the process.
  Prior to each interview and focus group, participants were informed about the study by reading and explaining the informed consent form. This was signed by each participant, leaving a copy in the possession of each one.
(iv) Stage 4: Communication of results. The results were presented to the participating communities, who also validated them. To ensure the validity and define the scope of the data construction techniques, the principles of saturation, saturation by technique and author, and triangulation by technique and author were applied.

The research was carried out within a framework of ethical protection, following bioethical deontological principles and the legal regulations in force in Chile.

The analysis process was carried out with the support of Atlas.ti 22 software, which facilitated the organization and systematization of the data. An analytical framework based on intersectionality was constructed, considering how factors such as age, gender, ethnicity, and rural context shape the food security experiences of the Mapuche elderly population. In addition, the interpretive-symbolic approach was incorporated to understand the construction of meanings from the perspective of the participants. To ensure the validity and rigor of the analysis, the preliminary results were presented to the participating communities in feedback sessions, in which the interpretations were contrasted with their experiences and perceptions, thus allowing for a process of community validation. In this way, the interpretation of the data was not limited to the researcher’s analysis but integrated the perspective of the participants, strengthening the legitimacy of the findings. In addition to the community validation, an instance of socialization and analysis of results was carried out with key institutional actors, specifically with regional managers and those in charge of programs of Agricultural Development and the Health Directorate. The purpose of this meeting was to strengthen the dialogue between scientific evidence and the formulation of public policies, allowing the contrasting of the findings of the study with the experience of those who design and implement programs aimed at food security in the rural Mapuche elderly population. From a methodological perspective, this instance is framed within the strategy of triangulation of sources [117] by integrating multiple stakeholders in the analysis and validation process. It also responds to an applied and participatory research approach [121] by not only documenting the problems identified but also generating inputs that can be used to improve public policies. The inclusion of these actors allows contextualizing the findings in the framework of food governance and intersectoral management, facilitating the identification of viable strategies to strengthen food security in rural Mapuche communities.

## 4. Results

The results obtained about the specific objectives proposed are presented below.

### 4.1. Problems Perceived by the Subjects Concerning Food Security in Boyeco’s Territory

**Figure 2:**
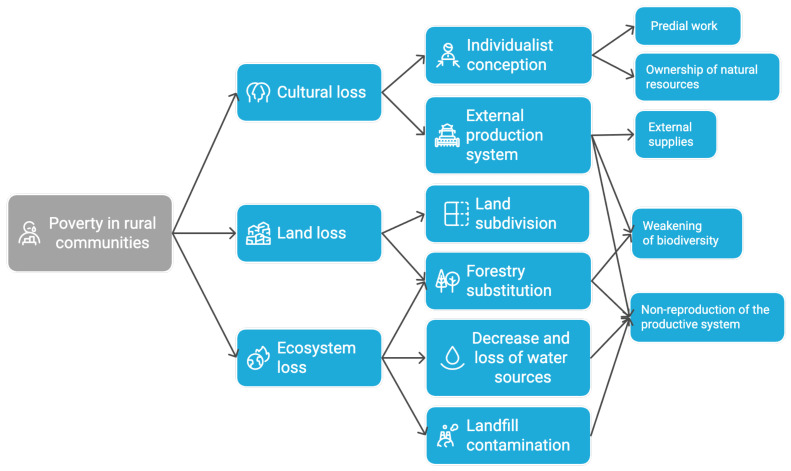
Subjects perception of poverty in rural communities.

A first finding is the interpretative positions and discourses related to the roles of the actors. It is important to highlight, as observed during the development of this research, the different roles played by the interviewees since their paradigms construct their discourses. Thus, we have leaders of the territorial roundtable with a political role with respect to their territory who recurrently allude to the colonialist relationship of institutions and society and the impact of their actions on the territory; leaders of Indigenous communities who observe their territory with a predominance of economic and social aspects; and the elderly see it from the affectation of their daily life in terms of food and culture. These differences in the construction of the discourse reflect not only the diversity of experiences and perceptions within the territory but also the way in which each group interprets and faces the challenges surrounding food security. Understanding these approaches allows for a more holistic view of the problem and underscores the need for policies that consider this plurality of perspectives for a more effective and culturally relevant intervention. From these differences in discourses and roles, various tensions and points of convergence emerge in relation to food security, which are presented below in Figure 2.

When asked about the problems of food security, the older rural Mapuche participants recalled how the family production system that they knew was reproduced, centered mainly on family consumption and the use of internal resources of the family unit and the community, and how this was being modified, given the alteration of production systems, community culture and the loss of land and the environment, especially after the arrival of the landfill in the territory, which had a very negative impact on the productive, social and cultural aspects.

The concept of ‘earth power’ was a concept that emerged describing the non-use of fertilizers; other people described the use of farmyard manure; never synthetic manure. In addition, the productions were sufficient for the central objective, which was family consumption.

On the other hand, the communal or collective conception allowed the sharing of natural resources, especially water, which at that time was not lacking, accessing lagoons, springs, and rivers that were available in the community, as well as the communal work of repairing roads and accesses, and productive support. This was because people worked in their fields and had the cultural conception and time for collective work; they also had a certain level of sovereignty over the use of their lands, which were more extensive and subsequently became smaller, both due to the increase in family and population size and the loss of land.

This is reflected in the testimonies that can be seen in Table 1.

Thus, the cultural loss, caused by the individualistic conception of the dominant culture, both in terms of agricultural labor and the ownership of natural resources, combined with the obligation imposed by public policies to use a culturally foreign production system with external inputs that kept increasing in cost, weakened biodiversity and the land’s resilience. This had a devastating effect on the reproduction of the productive system, which led to the poverty of rural families. This, along with the loss of land and ecosystems, especially due to reforestation with exotic species and invasive projects such as the landfill that polluted the territory, further eroded the environmental resilience and the families’ ability to live off the land.

People’s health was also affected, as the loss of families’ productive capacities and wage labor in the cities led to changes in food consumption, from preferentially consuming their own local production to relying on processed and ultraprocessed products.

**Figure 3:**
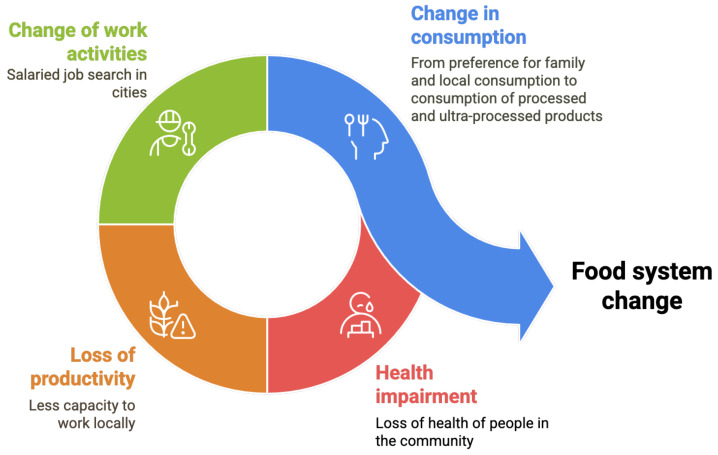
Subjects perception of changes in food system.

For all the reasons mentioned, people believe they have lost their food security (see Figure 3).

### 4.2. Perception Regarding the Implementation of Public Policies Related to Food Security in the Territory

When inquiring about programs related to food security, the participants identify state-run agricultural development programs, the public health system, and the public education system.

#### 4.2.1. Agricultural Development Promoted by Public Institutions

Participants observe that the criteria of the programs supporting family farming in the territory do not align with the production objectives for food security. This is because they perceive that the priorities of the productive support programs implemented by the National Institute of Agricultural Development (INDAP), the National Corporation for Indigenous Development (CONADI), and municipalities, such as the Indigenous Territorial Development Plan (PDTI), Rural Development Program (PRODER), and Local Development Program (PRODESAL), focus solely on commercialization and income generation, without integrating the family’s food consumption. Production is valued for yield rather than the quality of the food; monoculture production is supported, and chemical fertilizers and pesticides are applied. These perceptions are linked to the lack of cultural relevance regarding production methods and technical assistance based on the use of agrochemicals, which is inconsistent with their perception of healthy food. They also mention individual work without incorporating collective methods for community members, as well as the disconnection between the productive needs of families, which are not uniform. Additionally, they highlight the lack of necessary resources to achieve a productive goal, as the amounts provided are small. In this regard, they pointed out (see Table 2):

The lack of relevance between public policies and the foundations of what communities consider healthy production that meets their needs is an obstacle to food security. To strengthen this aspect, productive criteria would involve a preference for self-consumption and not just commercialization, with inputs that do not contaminate food or jeopardize health and with diversified and sufficient resources to achieve a positive effect on food security. In addition, culturally appropriate technical assistance.

They value the adjustments that the PDTI has made in recent years in the territory, which incorporates a diversity of productive sectors and their respective technical assistance, more aligned with the productive and food needs of the families, such as agricultural and livestock production, the incorporation of polycultures, with greater suitability and flexibility to the needs of the farms, according to their condition.

However, there is a turning point that can be seen in the words of the leaders during an assembly in which public institutions and leaders of the territorial roundtable and the communities participated: “We have a damaged territory with a myriad of situations that have not been addressed. Eradicating the landfill from the territory, which has a spiritual and physical impact, is imperative”. In response to these words, many leaders nod in agreement. This indicates how important it is to comply with the landfill closure plan and the process of territorial recovery.

#### 4.2.2. Food Security and Its Link to the Health and Education Systems

The participants identify what they perceive as deficiencies in the system. In terms of health, they refer to the primary healthcare system for older adults at the local Family Health Center (CESFAM) and the Adult Complementary Feeding Program (PACAM). In education, they focus on the School Feeding Program (PAE) implemented by the National Board of School Aid and Scholarships (JUNAEB).

Regarding health, they view the biomedical system as insufficient, focusing solely on disease recovery rather than disease prevention and health promotion. Moreover, disease recovery is carried out through medication without considering that environmental factors and current dietary dynamics are the main causes of many prevalent illnesses.

As for the Adult Complementary Feeding Program (PACAM), opinions are divided. Some see it as beneficial for those experiencing food insecurity. However, most participants believe it is not a suitable solution overall, as it consists of processed food products, which they find unappealing and unacceptable. Additionally, it does not align with their understanding of a healthy diet. For them, healthy eating means consuming ingredients produced at the family and local levels, prepared according to local traditions, with a particular emphasis on soups and stews.

The above can be observed in the testimonies presented in Table 3.

A system of health linked to the culture of the territory and the traditional Mapuche health system is proposed, addressing the conditions of the environment, including cultural, social, and ecosystem aspects. The food support should not be based solely on PACAM but rather promote systems of healthy food production, especially suitable gardens for older adults, and encourage cultural practices that value Mapuche culture.

Regarding the educational system, participants perceive that the food in schools is not good because it is not healthy. It is not linked to local production or cultural dietary patterns. One interviewee reflects, “Knowledge has to do with everything, with the body, with food, with nature; food is not only eating but also the interaction with nature, with the world and other species. It is the relationship with harmony, without abusing what is there”.

While they commented on some cultural education initiatives, these have not been consistent over time. Another person notes with regret, “Young people like my daughter are used to feeding themselves without considering their culture, and so they feed their children”. They propose that schools should incorporate culture into all their areas, including food, offering healthier foods, and incorporating traditional ones (see Table 4).

The participants propose that the PAE should improve, being consistent with the territorial production and cultural dietary patterns. Contributing to territorial development with local supplies, promoting local culture and ancestral food culture from production to consumption.

### 4.3. Proposals to Address the Issues Raised

The majority sentiment of the participants reflects the idea of restoring the sense of security, satisfaction, and dignity of older adults through the garden, which allows them to have a role in their care, in addition to sharing and contributing to their family members and neighbors by producing something healthy. A leader reflects, “Older adults have grown up with vegetable gardens. That gives their lives meaning, and they take shelter in that, emotionally”. Having a garden is also associated with well-being; although many older people have certain illnesses, this would not prevent them from performing some daily tasks, with help available for the heavier or more laborious ones. This is expressed in Table 5.

### 4.4. Proposals for Change

After discussing the state of food security in their communities and territory, they observe that it is possible to modify and recover some of what has been lost, taking into account both structural factors and local initiatives that can be implemented. Some families have made individual progress in certain initiatives within their units, especially regarding forest naturalization, but they mention that much more is required, and public programs could contribute, always in a participatory manner, with the communities and territory (see Table 6).

From the perspective of the viability of recovering culturally appropriate food production and ways of life, land recovery is seen as an essential factor for revitalizing culture, well-being, and the sustainability of productive practices, which include polyculture farming, livestock farming, poultry farming, and forestry. Another fundamental factor relates to being respected and not violated by projects that intrude on their spaces and ways of life, such as the implementation of the landfill that was established in the territory for more than two decades, which had a negative impact on social and community life and the loss of productivity in the fields.Another leading member firmly reflects, “The diagnoses and proposals are there, but as [the way] in which wealth is distributed in Chile, we have to aspire for the cake to be well distributed. This inequity also produces food insecurity”.

To achieve the above, it is necessary to restore the capabilities of the territory through the recovery of forests and water, the implementation of proper water collection or harvesting systems, and traditional and ecological productive practices that contribute to food security for older people and communities in general, in alignment with food culture, which is especially relevant in the context of rural Mapuche elderly people.

For this, they admit that public policies can play a significant role as long as they are adapted to a technology transfer that respects them and is relevant to their culture, with participatory and culturally and territorially validated methodological practices.

## 5. Discussion

The results of this study reveal a disconnection between public food security policies and the sociocultural reality of rural Mapuche older adults. Current programs maintain criteria of the green revolution and have not been sufficiently open to new productive models more linked to the culture of the territory, adopting a mostly productivist approach without considering the needs of self-consumption or the preservation of traditional practices. From the participants’ perspective, traditional production and consumption systems have changed significantly, and the main barriers to their recovery are related to land reduction, environmental degradation, and the imposition of external production models. These factors have not only affected food security but have also weakened community ties and the transmission of ancestral knowledge.

The same occurs with the public health and education systems, whose orientations give very little room for integration with the culture of the contexts in which they are inserted. These mentions are consistent with the views of other authors [3,98].

These findings coincide with previous research that shows that food insecurity in Indigenous communities is not only a question of scarcity of resources but essentially a matter of structural barriers that have been maintained and have affected public policies, which fail to adjust to local cosmovisions and traditional practices [122,123,124]. Research in other Indigenous communities across Latin America has indicated that the forced transition to agricultural systems reliant on external inputs has led to a decline in biodiversity and reduced the resilience of rural Indigenous communities in terms of food security [125,126]. The case of rural Mapuche older adults in this study confirms this trend, highlighting the urgency of designing interventions that incorporate cultural and environmental dimensions into food security policies.

It is observed that government programs tend to homogenize intervention strategies without considering the socio-economic and cultural particularities of Mapuche communities. For these groups, food insecurity is not limited to physical access to food but also involves the loss of autonomy and dignity in its production. The promotion of policies that favor monoculture and the use of agrochemicals conflicts with their traditional principles, generating a sense of uprootedness and greater economic dependence. Likewise, the exclusion of traditional knowledge from state programs reinforces a perception of marginalization and subordination, affecting social cohesion and community well-being.

The results also suggest that the healthcare and education systems do not effectively address food security in this age group. In healthcare, the treatment of chronic diseases is focused on medicalization rather than prevention, fostering dependence on pharmaceutical treatments that are often difficult to access instead of promoting healthy eating habits. In education, the disconnect between school meals and local food production represents a missed opportunity to strengthen food sovereignty and the intergenerational transmission of knowledge in Mapuche communities.

Integrating successful models, such as Brazil’s PNAE and market-access initiatives in Cameroon, into food security policies could help address these challenges. Prioritizing local procurement, small-scale farming, and strengthening market access, as seen in these cases, would support the autonomy of rural Mapuche, older adults while preserving traditional agricultural practices and strengthening food sovereignty [61,127]. Even so, these policies face challenges in promoting sustainable agriculture, as many mechanisms continue to favor conventional production systems [128]. There remains a need for increased investment in existing programs and a shift towards more sustainable agricultural practices to ensure the long-term viability [128,129].

The need to reformulate public food security policies in Indigenous communities is evident. A participatory approach is required, one that includes local actors and representatives of this demographic group in the design and implementation of strategies, integrating ancestral knowledge with relevant technological advancements. The promotion of home gardens, the recovery of water sources, and the restoration of the traditional agricultural landscape emerge as key strategies to reverse the current state of vulnerability.

### 5.1. Limitations of the Study

This study presents some limitations that should be considered when interpreting its findings. While the ethnographic methodology allowed for an in-depth understanding of the participants’ experiences and perceptions, the research focused on a specific territory in the Araucanía Region, which limits the generalizability of the results to other Mapuche communities or Indigenous peoples with different geographical and sociocultural contexts.

### 5.2. Future Investigation

This study opens multiple lines of research that can enhance knowledge about food security in rural Indigenous communities. First, it would be relevant to conduct comparative studies among different Mapuche communities, as well as with other Indigenous peoples in Chile and Latin America, to identify patterns and differences in perceptions of food security and the effectiveness of public policies based on geographical regions.

Additionally, incorporating quantitative approaches could complement qualitative findings and measure the impact of policies on food security. Analyzing the evolution of production and consumption systems over time would allow for a better understanding of the dynamics of change and the factors that have influenced the transformation of these demographic groups’ dietary practices.

Finally, it is recommended to explore the role of community networks and territorial organizations in mitigating food insecurity. Research on successful local initiatives could provide replicable models for designing more inclusive and effective policies, promoting food self-sufficiency, and strengthening cultural identity.

## 6. Conclusions

This study has demonstrated that public food security policies targeting rural Mapuche older adults lack cultural and territorial adaptation, deepening the perception of food insecurity in this population. The disruption of production systems, environmental impact, and the forced adoption of external agricultural models have weakened traditional practices, compromising both the quality of nutrition and the social and cultural well-being of these communities.

Since the existing programs in Chile are either general in their target population or do not directly address food security, it can be stated that there are no permanent food security development programs specifically aimed at rural Mapuche older adults. This represents a significant gap in current public policies, as they fail to meet the needs of this group by prioritizing commercialization over self-consumption and inadequately integrating traditional knowledge. By centering the perspectives of rural Mapuche older adults, this study highlights how food security is not only a matter of access but a fundamental element of cultural preservation, intergenerational knowledge transmission, and autonomy. This underscores the urgent need to reformulate state strategies to incorporate participatory and culturally relevant approaches, where local production and traditional practices are valued as fundamental elements of food security. Additionally, public policies should adapt to the context of an aging population with practices that integrate and contribute to their well-being.

The findings indicate that food security in this context cannot be addressed solely through a productivist or welfare-based approach; it must integrate autonomy, cultural identity, and environmental sustainability. This study contributes uniquely by demonstrating that the misalignment between agricultural development policies and the Mapuche cosmovision not only affects food availability but also erodes communal ties and weakens resilience. For the participants, access to food is not sufficient if it does not come from safe, culturally significant, and long-term sustainable sources. The misalignment between agricultural development policies and the Mapuche cosmovision has led to increasing market dependency and a loss of food sovereignty, ultimately weakening the community’s resilience.

The way in which the people interviewed conceptualize food security is deeply influenced by their roles and experiences within the territory. Some emphasize power relations and territorial autonomy, others prioritize the economic and social dimension, and the elderly situate food as an axis of cultural continuity and daily well-being. These differences underline the importance of multidimensional approaches in the analysis and implementation of public policies in the territory.

Finally, the study highlights the importance of promoting intervention models that strengthen food self-sufficiency through gardens, land recovery, and equitable access to natural and cultural heritage. Unlike previous research that primarily focuses on economic and nutritional indicators, this study underscores the essential role of cultural identity and traditional knowledge in shaping perceptions of food security. Only through a comprehensive and respectful approach to Mapuche culture can we advance in the development of effective, equitable, and sustainable public policies, ensuring food security for present and future generations.

## Figures and Tables

**Table 1:** Loss of the traditional Mapuche production system.

Participant	Testimony
HM1	‘…before, no fertilizer was used in the fields, no fertilizer or anything else, just like that, you fallow, cross, sow and harvest 80, 60 or 100 sacks of wheat.’
HM2	‘…in those days you ate the wheat, you didn’t put anything in it, pure earth power, the beans, all those things, earth power, so they didn’t have any of those chemical things.’
DH1	‘Before, we lived in a community, and whoever had water, there was no problem for us to go and fetch water there and drink from that place, but not now, now they are private places, so we see ourselves in a way… we have fallen into the system itself, we cannot fetch and dispose of the same things that are in the community.’
GF2	‘A neighbor was telling me that before, I don’t know, people would come together to fix the roads because they lived here, they had the time, and they owned their time…’
DM2	‘…before, in the *collihuin* [collective work], which in the past was done by sowing everything collectively, one helped others, and so we helped each other with the harvests. The little was the same because there was not so much that could be sown, but something more was sown than now.’
DM3	‘After the 1990s, people started to fence in a little bit at a time before there was nothing. It was just free fields and below and without many houses as well.’
MM1	‘A long time ago, when there weren’t so many eucalyptus trees, we had a water dam. We used to irrigate with a water motor, where I was telling you, and we irrigated with a water motor, and now, even if you have a motor, you have nowhere to get water. That’s the problem.’
GF2	‘The other is that the fields are getting smaller and smaller, because when we used to talk about 20, 30, or 40 hectares that belonged to the fathers of the fathers, then they got smaller and smaller, and now we find ourselves with plots that are two and a half, three and a half, and those who have a lot have five, and it turns out that you have to deduct the space of the house from that, so there is less and less capacity to reproduce and generate income, which is why people go out to work.’
DM1	‘That way of producing or of production was what effectively meant that the ancestors had a *kimun*, a way of thinking, a special wisdom, and where the food, apart from being healthy, had volume, there was quality, mainly quality in the products. So I think that for me that is what food security should be all about.’
DH1	‘…now to have something organic, we would have to have the animals that we no longer have to make the compound to be able to fertilize organically, so we have all these factors that in some way make it very difficult for us to be more competitive in this aspect. We can, but in small quantities.’
HM1	‘The *Yuyo* [plant] was eaten, now there is no *Yuyo* because the fields are being fumigated, they are getting spoiled, the *Yuyo* has been killed. Of course, the land has been spoiled by fumigation, and now the wheat has also been contaminated because there is a lot of fertilizer, because we have to fumigate to sow, we have to fumigate to harvest.’
DH1	‘…we required a certain amount of food for the year, and at first, it was difficult! But we would reach the goal. As time went on, we started falling behind, like with 80%, and we ended up at 50% of production versus consumption… then the children started arriving, and we had the need to look for other horizons, like going to the city to seek better income.’
DM2	‘And especially in young families, because the productive aspect is no longer there, especially in the Boyeco territory, due to the water scarcity, water pollution, and also because people have neglected, in this case, the Mapuche, the natural production aspect, as the animals that produced manure are no longer around.’
DM1	‘*Chuta* [exclamation], at what point can we make changes to this? Because here, with that kind of violence, they contaminate the waters, invade you with trash, put in hydroelectric plants, wastewater treatment plants, and a countless number of things that, while it’s true they might be related to how the city develops, it comes at the expense of those of us who live on the outskirts.’
DH1	‘…and then we started consuming processed foods, and that’s already altered. It also alters our bodies. Therefore, here, in the city and in the countryside, you didn’t see so many people with diabetes…’
DH1	‘If we stay working here in the countryside, maybe my answer will be a little harsh in some way, but we’ll be scratching by because the countryside doesn’t give us enough to live, it doesn’t give us enough anymore!’
DM1	‘So, what kind of food security could we talk about here if we’ve been stripped of our own ways of life?’

**Table 2:** How participants view the agricultural development programs promoted by public institutions.

Participant	Testimony
DM1	‘…we have seen in our own communities how public policies from INDAP, from agriculture [the Ministry of Agriculture], keep insisting on programs like PDTI, PRODESAL, and PRODER, where the only thing they explain to people is that they have to produce, and hopefully a lot, and in my opinion, this emphasis on quantity over quality doesn’t make sense.’
DM3	‘The PDTI has been changing, but the others haven’t because they are very structured and were based on monocultures. If someone didn’t adapt, it was considered a failure, and they would say: ‘of course, so much has been invested in the communities, but the people aren’t thriving.’ But it was because they were changing the way people saw and thought about things, so that’s why it led directly to failure.’
DM2	‘The municipality with its PRODER, PRODESAL programs, in order to progress, and all the others started, for example, working with the family, but in an individual manner, and some with microenterprises, where you couldn’t even have pigs, chickens, ducks, or anything, because you had to focus solely on one line of business…’
DH1	‘They help a little bit with this and a little bit with that, a little bit with production and a little bit with livestock, and always just a little bit. It’s like they give people a candy but not real, long-term solutions. Because the PDTI, in this case, to refer to that topic, offers an annual bonus every so often of 100,000 or 200,000 [USD 100 and USD 200, respectively], and what do you do with that kind of bonus?’
GF1	‘…several requests for land subsidies have been made, but they haven’t been successful.’
DM3	‘Before, it was like people were forced to build huge greenhouses because those were worth it, and it wasn’t thought like that, in terms of the family’s economy, the family’s food, but always focused on sales.’
GF1	‘Fertilizers are what don’t convince much; the soil analysis is good, but after that, a more natural product should be used.’
DM3	‘…so the technicians and the people who came from the support programs came to see how much money had been earned, but the people didn’t worry so much about that. They cared more about feeding themselves, and that wasn’t taken into account.’
GF2	‘…we had previous experiences when we planted garlic down there, we planted community peas, we had a breeder pig over there, haha, and it never worked out. Why? Because a few people worked, and the others stopped coming, and then when it was time to harvest, those were the first ones to show up… of course, since it’s community-based, it’s for everyone.’
GF1	‘…here we’ve always worked with strawberries, peas, and green beans, and then when you want to get an irrigation subsidy, they say no, you can’t, and then they start to backtrack. But later, when they wanted us to support them, that’s when it was possible.’
GF2	‘To get the greenhouse, you have to apply for INDAP projects, and they set their conditions. You have to be INDAP users, and to be INDAP users, you have to justify 50% of the production from the field, and the other 50% can come from elsewhere…’

**Table 3:** Primary health care system in public health.

Participant	Health System
DM1	‘Because here they act only when you are already sick; they don’t act based on how we can actually prevent diseases. With healthy cultivation methods, we can prevent diseases. With healthcare approaches that consider ancestral medicine, we can work on disease prevention. And by maintaining a healthy lifestyle, with clean and pure water, we can also prevent illnesses.’
DM2	‘…I was born in ’55, and since the ’60s, as far back as I can remember, people used to farm without fertilizers, and they weren’t as sick as we are now. Nowadays, we all leave the clinics with a ridiculous amount of medication. It makes me wonder—are we all sick with the same thing? Because it really catches my attention that people walk out with 8 to 10 different prescriptions. Honestly, I think we need to look back at past agricultural practices.’
GF2	‘…the older adults, they go to the clinic for a cardiovascular check-up once a year! And they’re given a prescription with all the medication for the entire year!’
DH1	‘…In the countryside, people are no longer producing what they used to in order to have a better quality of life and a healthier diet, so it’s very complex to simply say, ‘Look, grandma, eat healthy.’
DM1	‘The PACAM provided to older adults is a great contribution. It’s a help for those who have nothing, but it cannot be the solution! It’s just a temporary fix. What they should do is promote gardens that are suitable for older adults.’
DM3	They have no trust in [referring to PACAM cream soup], even though it might be easier to prepare, and sometimes, due to dental issues, a creamy soup could be more convenient for them. But if they have the choice, they prefer eating a *cazuela* [traditional Chilean soup], even if it’s just a simple soup. ‘Now this is food,’ they say. ‘Now my body feels right; this is what I needed.’
DM3	‘…the bags they are given at the clinics, that’s what causes their resistance to taking those processed food bags.’
MM4	‘Maybe they should give them more milk, because for example, now they don’t give them milk, they give them milk substitutes, which isn’t milk, it’s a dairy drink, and it’s bad. You add water, and it curdles, and what elderly person is going to eat that if it’s not good? I say this because we have elderly people next door who are given that, and that milk is really bad, and the grandfather, if he’s alone, isn’t going to eat that.’
DM3	‘…there are other elderly people who are also treated here at the CESFAM and receive those creams given by the elderly nutrition program, but they are not well received, at least from what I’ve seen up close with family members.’
DM3	‘…the food is mainly a soup, that’s like the good food, a lot of toasted flour, seasonal vegetables, that’s generally what they think of when talking about food, at least; because, for example, a grainy rice is not considered a good lunch.’

**Table 4:** Public education system.

Participant	Public Education System
MM4	‘I think it should start primarily with food in schools because it is very bad, now they are trying to improve it a little. But I think the food should try to go back a little to the past, especially for rural children, and if it’s at a national level, it would be better too.’
DM3	‘I asked a girl at school why she didn’t eat the lunch, even though it was nutritious and all, and she told me that it wasn’t food. For her, she would eat the soup.’
MM4	‘…there’s work to be done, JUNAEB has to improve the food, make it more natural, more local.’
MM1	‘I was in a school for 3 years teaching the children a bit of Mapuche. I would go and knit, spin. I left about three rugs in the school. I would knit, and the children would watch me. ‘How do you do it, aunt?’ they would ask. Spinning everything, winding thread. I made *mote* [generic term for varieties of boiled grains], toasted flour, and I also made *catutos* [traditional Mapuche preparation].’

**Table 5:** Food and the garden as a heritage of security and food sovereignty for older adults.

DM3	‘That’s what makes them feel good, and they feel proud because, for example, when you visit the elderly, the first thing they will show you is the garden. They proudly show their gardens and invite others. For example, they bring fresh new potatoes and share them with their families or neighbors. Then the other one brings something back, like new broad beans or peas.’
MM1	‘I plant everything when I’m feeling good: beetroot, chard, and this year, radishes. But, ma’am, they told me they were so red and beautiful. The price was really good until March. After that, I got sick, and my garden stopped, but now I have to work again.’
DM3	‘I see that this is more than anything about scarcity. They see it as if not having a garden and not having their little chicken means they don’t have anything.’
DM3	‘And people say it, they are aware that chemical products harm their health, so in some cases, there’s no choice but to use them, but preferably they look for what is grown here in their gardens or from their neighbors.’
MM1	‘I have a worker who comes to help me plant and pile up. I have to pay him. Pay him and provide food, that’s how I had him.’
DM3	‘And the other thing is that there are also elderly people who are relatively alone, so sometimes, due to their age and health issues, they are unable to tend to their gardens, and that limits them as well. You can see the suffering in that sense because they are used to having a garden at home.’
GF2	‘The advantage here compared to the old times is that now almost all elderly people have a pension, whether it’s the basic assistance pension or a little more, but with that, it helps them a lot to buy supermarket food or other items.’

**Table 6:** Proposals.

DM1	‘Why do the Mapuches fight for the land? Because it has been usurped for centuries, and today the land is where we live, it is our *Ñukemapu* [Mother Earth], it is our mother. So today, we must try to recover spaces so we don’t live in such overcrowded conditions and, primarily, respect the old ways of life that were connected to nature. So, here in the territory, at least in Boyeco, we have said that a space has been broken, a space that we carry with us, related to the *iltrofilmognen* [good living], and this has caused not only harm to those ways of life but also to us, as humans, as people, as Mapuche, who have lived in this environment. There have also been other types of illnesses related to psychological, physical, and especially spiritual diseases.’
DM2	‘…Many institutions support the idea that we had no right to demand the closure of the dump because we lived farther away from the place. The thing is, my place is 3 km away in a direct line, but I still had the flies, the smell, the packs of dogs, everything. So, it’s not just the space where the trash is located… it spans an area of almost 15 or 20 km around it. It took a long time, a really long time, but eventually, people realized that the dump had been harmful, but it took a long time.’
DM1	‘That no more places where Indigenous communities exist would be abused and run over with these types of constructions, like sewage treatment plants, like the dump that is mainly in Indigenous communities. And why in Indigenous communities? See… because they see us as second or third-class citizens!’
DM3	‘Let’s see, the water, the health of the people, whether they are alone or not, what else could it be, the quality of the land also influences because, for as long as I can remember, families used to worry about maintaining the quality of the soils by incorporating organic manure from the animals…’
HM3	‘Also implement water conservation projects, so that the government provides these projects to the communities and doesn’t make it so bureaucratic, having to go through a lot of procedures just to apply for a small water pool.’
GF1	‘Here, a lot of trees like eucalyptus and pine have already been removed. Because, in reality, the water keeps draining away, and there’s not much water left, so that’s why those plants have been removed and replaced with native ones in the end.’
DM1	‘Not being predators, and not being invaders, like what happens today with the forestry companies, leaving behind a number of species that disappear and become extinct [referring to native species], and they take away the water.’
GF1	‘It should be vegetables that one grows without chemicals. It should be natural, something like going back to the old days when we planted without chemicals and had good food that was full of vitamins.’
GF2	‘I’m crossing my fingers that we get to 40 h [referring to Chilean legal weekly working hours], so we can work from Monday to Friday because then, at least with two days off, Saturday and Sunday, one could do something more at home.’
MM2	‘And one has to know how to get along with people, ask because not all communities are the same; the president says, ’Okay, let’s buy poles, everyone gets poles, all the same!’ But I don’t. I look at the person’s needs because I don’t gain anything from buying poles for someone if I know they already have everything fenced.’
DM1	‘…a demonstration space for cultivating all the food that one considers much healthier, because that way still exists and I believe it should be recovered a bit, this exchange of knowledge, because we have so much knowledge, we have *lawentuchefes* [Mapuche doctor specialized in the knowledge, preparation, and application of herbal medicine], we have people dedicated to cultivating different products, we have people who do crafts, I mean, we have an infinity of resources where we could be somewhat self-sufficient, but at least for self-consumption.’
DM1	‘There need to be deep changes, and these deep changes must come from the institutions, but also, with the proposal from here, what we want to be done in the different spaces and territories, how we see the issue of health today in all aspects and how this issue of toxins, chemicals, adulterants, and new forms of production affects us. I believe there are pending challenges, but above all, the institution must consider this way of life that is being threatened by a capitalist system that only wants volume, market, and money.’
DM1	‘…but I believe that the food should be different, it should be healthier, it should be more flavorful, it should be colorful. It should be something much more natural, let’s say, if it’s an apple, it should be an apple, but not something in a package, in powder form, you see? Because it’s not an apple, it’s not a pear, it’s not a real fruit. Why does it have to be linked to things that the elderly people here didn’t know in their time? So they say no, no, no, I don’t like it. It might have a thousand properties, but the taste won’t be the same as the other one. So I think this is a bit of a call from public health…’

## Data Availability

The original contributions presented in the study are included in the article, further inquiries can be directed to the corresponding author.
